# Delay analysis of pulmonary tuberculosis in the eastern coastal county of China from 2010 to 2021: evidence from two surveillance systems

**DOI:** 10.3389/fpubh.2023.1233637

**Published:** 2023-08-11

**Authors:** Kui Liu, Rui Ge, Dan Luo, Yan Zheng, Zhenye Shen, Bin Chen, Wei Feng, Qionghai Wu

**Affiliations:** ^1^Department of Tuberculosis Control and Prevention, Zhejiang Provincial Center for Disease Control and Prevention, Hangzhou, Zhejiang, China; ^2^Department of Tuberculosis Control and Prevention, Jiaxing Center for Disease Control and Prevention, Jiaxing, Zhejiang, China; ^3^Department of Public Health, Hangzhou Medical College, Hangzhou, Zhejiang, China; ^4^Fenghua Center for Disease Control and Prevention, Ningbo, Zhejiang, China; ^5^Taizhou Central Hospital (Taizhou University Hospital), Taizhou, Zhejiang, China

**Keywords:** pulmonary tuberculosis, delay, logistic regression, surveillance systems, older people

## Abstract

**Background:**

Tuberculosis (TB) remains a major public health challenge. However, indicators of delays in assessing effective TB prevention and control and its influencing factors have not been investigated in the eastern coastal county of China.

**Methods:**

All notified pulmonary tuberculosis (PTB) cases in the Fenghua District, China were collected between 2010 and 2021 from the available TB information management system. Comparison of delays involving patient, health system, and total delays among local and migrant cases. Additionally, in correlation with available Basic Public Health Service Project system, we performed univariate and multivariate logistic regression analyses identified the influencing factors associated with patient and total delays in patients aged >60 years.

**Results:**

In total, 3,442 PTB cases were notified, including 1,725 local and 1,717 migrant patients, with a male-to-female ratio of 2.13:1. Median patient and total delays of local TB patients were longer than those for migrant patients; the median health system delay did not show any significant difference. For patient delay among the older adult, female (cOR: 1.93, 95% CI: 1.07–3.48), educational level of elementary school and middle school (cOR: 0.23, 95% CI: 0.06–0.84) had a statistical difference from univariable analysis; however, patients without diabetes showed a higher delay for multiple-factor analysis (aOR: 2.12, 95% CI: 1.02–4.41). Furthermore, only the education level of elementary school and middle school presented a low total delay for both univariate (cOR: 0.22, 95% CI: 0.06–0.82) and multivariate analysis (aOR: 0.21, 95% CI: 0.05–0.83) in the older patients.

**Conclusion:**

The delay of TB cases among migrants was lower than the local population in the Fenghua District, which may be related to the “healthy migrant effect”. It highlights that women, illiterate people, and people without diabetes are key groups for reducing delays among older adults. Health awareness should focus on these target populations, providing accessible health services, and reducing the time from symptom onset to diagnosis.

## 1. Introduction

Tuberculosis (TB), caused by the *Mycobacterium tuberculosis* complex, remains one of the leading causes of death globally (1). Approximately a quarter of the global population is infected, with only 5–15% developing active TB (2). National Governments have undertaken concerted efforts in TB care and prevention to achieve the United Nations Sustainable Development Goals and the World Health Organization (WHO) End TB strategy, resulting in a 3.4% reduction per year in mortality rate and 1.9% reduction per year in global incidence rate (3). Despite the prevalence of the coronavirus disease (COVID-19), 9.87 million newly diagnosed TB cases worldwide have been reported, with an incidence rate of 127 per 100,000 in 2021 (4). Furthermore, China, the second country affected by TB, confronted an additional crisis derived from stagnant TB incidence, with an annual decline rate that was only half of what it had been before 2015 (5). Therefore, effective and efficient reduction of TB prevalence remains an enormous public health challenge.

Passive case-finding (PCF) is an essential approach for identifying active TB in major national TB control programs; often only those with obvious or severe symptoms of pulmonary TB (PTB) actively seek healthcare services (6). Several factors might influence the implementation of PCF, such as health-seeking behaviors, physician competency, and laboratory diagnostic capability, which could cause inevitable delays (7). Furthermore, delays in diagnosis and treatment could negatively impact prognosis and result in increased TB transmission in the general population, especially in low- and middle-income countries (8, 9). Thus, timely identification of factors influencing delays and ways to reduce delays would aid in optimizing available TB prevention and control strategies, with focus on eliminating TB (10).

In this study, we aimed to collect and present data from Fenghua District in eastern China to identify the delay characteristics of patients with TB. Additionally, an excellent health information system from Basic Public Health Service Project (BPHSP) has been established and operated for decades in this region, which could provide more precise portraits of patients with delays, including lifestyle, to implement further control and prevention strategies.

## 2. Methods

### 2.1. Overview

**Figure 1:**
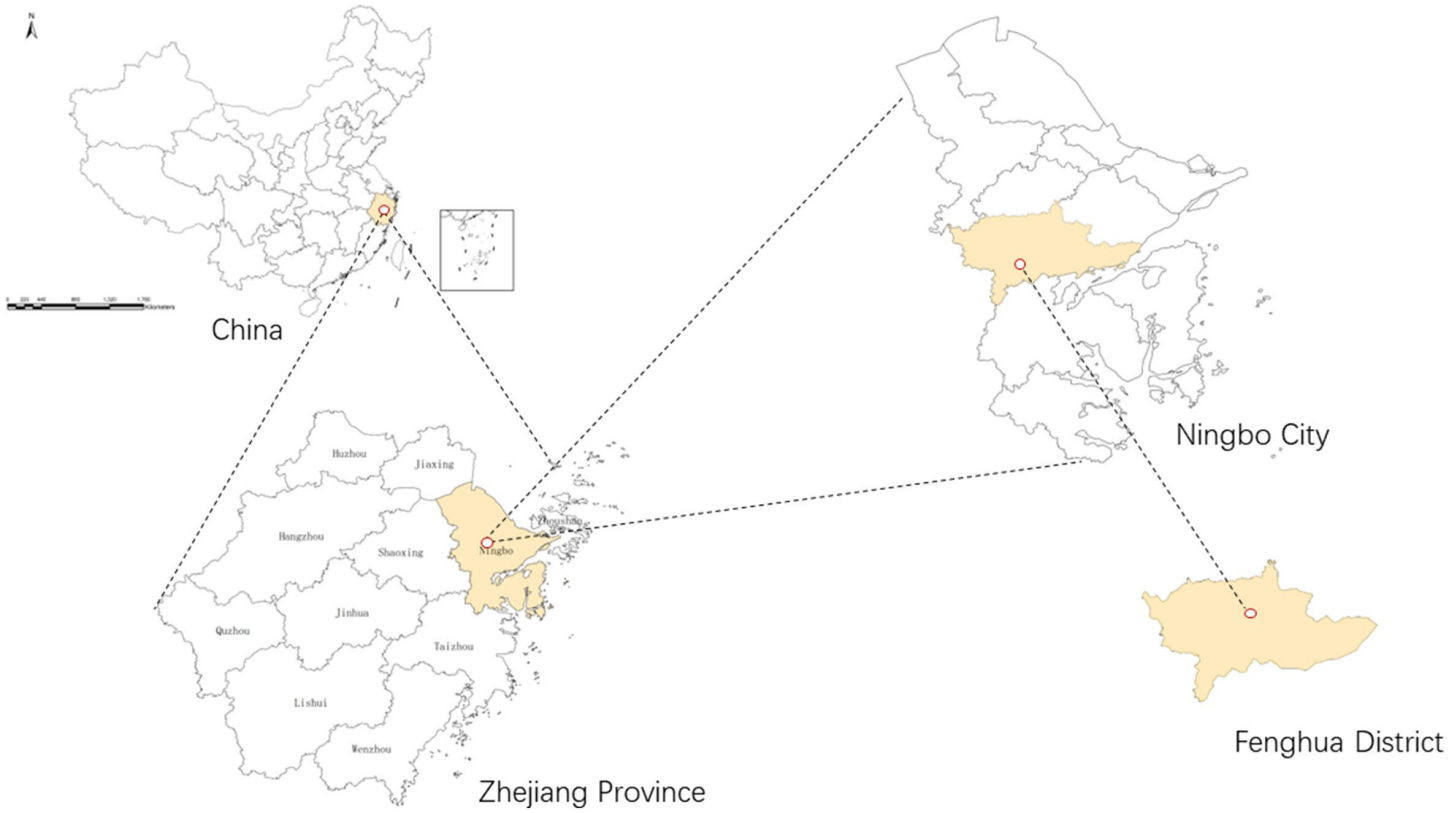
The location of Fenghua District.

Fenghua District, located in the eastern area of Zhejiang Province, belongs to the Ningbo Municipality. It comprises eight communities and four towns, with an area of 1,253 km^2^. Based on the seventh population census, Fenghua District had a permanent population of 577, 505 with approximately 135,568 aged >60 years, accounting for 23.47% of the population. The location of Fenghua District is shown in Figure 1.

### 2.2. Data collection

All notified PTB cases in Fenghua District were collected between 2010 and 2021 from the available TB information management system (TBIMS), a nationwide network reporting system in which all presumptive PTB cases are diagnosed and compulsively notified (11, 12). This system provides details, including demographic information, diagnosis, laboratory outcomes, and treatment outcomes of all notified cases. Furthermore, the BPHSP system was also constructed in Ningbo Municipality. With this, all residents aged >60 years were provided with voluntary annual health checkups. Additional details such as dietary habits, smoking status, and drinking habits were recorded using this system. We matched cases of TBIMS with BPHSP using a uniform identity card number to identify the relevance of various delays.

### 2.3. Definition

All PTB cases were laboratory-confirmed and clinically diagnosed. Laboratory-confirmed PTB was defined as a diagnosis with bacteriological evidence through sputum smear, sputum culture, or eligible rapid diagnostic technology. Clinically diagnosed PTB was defined as a diagnosis with clinical symptoms, chest imaging, epidemiological clues, and other tests (13). The national diagnostic criteria for PTB (WS288-2008 and WS288-2017) and the classification of TB (WS196-2001 and WS196-2017) in China were referenced to identify PTB cases. We defined smoking based on three categories: “Never smoker,” “Prior smoker,” and “Current smoker.” Drinking status in the previous year was classified into: non-drinkers, occasional drinkers (≤1 a week), and frequent drinkers (>1 a week). The body mass index (BMI) values were classified into: <18.5, 18.5–24, and ≥24 kg/m^2^. Diabetes was defined as a prior diagnosis (collected through health records) or through a positive fasting glucose test. Health insurance included basic health insurance for urban residents and others. The former had a low reimbursement rate, and deficiency in both cap line and deductible line than other insurance.

### 2.4. Epidemiological characteristics of PTB and the delays

We analyzed the epidemiological characteristics of patients with TB in the Fenghua District. The variables included census register, sex, age, occupation, interval from onset to visiting a TB designated hospital, and interval from visiting a TB designated hospital to confirming PTB. Additionally, we compared patients, health systems, and total delays between local and migrant patients with TB. Patient delay was defined as the interval between the appearance of TB symptoms and first visit to a health facility. Health system delay was defined as the time between the first visit to a health facility and confirmed PTB diagnosis in a TB designated hospital. We used 14 days as the cut-off point for analyzing patient and health system delays and 28 days for the total delay.

### 2.5. Univariate and multivariate logistic regression analysis for delay

We matched 202 patients with TB aged >60 years from the TBIMS and BPHSP. We performed univariate and multivariate logistic regression analyses to identify the factors influencing patient and total delays. The influencing factors included age, sex, educational level, marital status, occupation, smoking status, drinking status, BMI, physical exercise, treatment category, diabetes, and bacteriological results.

### 2.6. Ethical approval

All included PTB cases were anonymized prior to further analysis. The Ethics Committee of the Zhejiang Provincial Center for Disease Control and Prevention approved this study. Given that only PTB surveillance data were used, the requirement for informed consent was waived. All materials used strictly followed the Law of the Prevention and Treatment of Infectious Diseases in the People’s Republic of China.

### 2.7. Statistical analysis

A descriptive analysis of demographic information was performed to present the general epidemiological features. Continuous variables were presented as medians and interquartile ranges, and categorical variables were presented as counts and proportions. To compare the patient, health system, and total delays among local and migrant cases, a Mann–Whitney U test was performed and a chi-square test was performed to compare the delay proportion. Furthermore, Joinpoint 4.9.0.0 software was applied to calculate the annual percentage change (APC) in TB notification rate from 2010 to 2021 to identify a trend. The location of Fenghua District was determined using ArcGIS software (version 10.2, SERI Inc., Redlands, CA, United States). Additionally, the R v4.0.5 software^1^ and SPSS Statistics (version 20.0; IBM Corp., Armonk, NY, United States) were used for the statistical analyses. *p* < 0.05 was considered statistically significant.

## 3. Results

### 3.1. Demographic characteristics of TB patients

In total, 3,442 PTB cases were reported in the Fenghua District from 2010 to 2021, including 1,725 local and 1,717 migrant patients. There were 2,343 male cases and 1,099 female cases, with a male-to-female ratio of 2.13:1. The occupational distribution was predominantly farmers, accounting for 51.57% of all the reported cases. Most cases were concentrated in the 15–59 years group, with a proportion of 74.20%. The notification rate was 57.53/100,000 in 2010 and 47. 97/100,000 in 2021, showing an overall decreasing trend (APC = −2.09%, *p* < 0.05). More details were displayed in Table 1.

**Table 1:** Demographic characteristics of TB patients from 2010 to 2021 in Fenghua District.

Characteristic	2010	2011	2012	2013	2014	2015	2016	2017	2018	2019	2020	2021
Total												
Number of cases	305	328	295	263	317	272	279	310	293	278	225	277
Incidence rate (1/100,000)	57.53	66.71	59.57	53.26	64.01	54.83	55.15	60.89	57.34	53.98	43.60	47.97
Census register, n (%)												
Local TB patients	183 (60.00)	181 (55.18)	187 (63.39)	162 (61.6)	165 (52.05)	143 (52.57)	135 (48.39)	148 (47.74)	122 (41.64)	121 (43.53)	80 (35.56)	98 (35.38)
Migrant TB patients	122 (40.00)	147 (44.82)	108 (36.61)	101 (38.4)	152 (47.95)	129 (47.43)	144 (51.61)	162 (52.26)	171 (58.36)	157 (56.47)	145 (64.44)	179 (64.62)
Sex, n (%)												
Male	207 (67.87)	223 (67.99)	182 (61.69)	170 (64.64)	213 (67.19)	179 (65.81)	201 (72.04)	208 (67.10)	203 (69.28)	201 (72.30)	159 (70.67)	197 (71.12)
Female	98 (32.13)	105 (32.01)	113 (38.31)	93 (35.36)	104 (32.81)	93 (34.19)	78 (27.96)	102 (32.90)	90 (30.72)	77 (27.70)	66 (29.33)	80 (28.88)
Age group, n (%)												
0–	2 (0.66)	4 (1.22)	8 (2.71)	0 (0.00)	2 (0.63)	1 (0.37)	2 (0.72)	2 (0.65)	2 (0.68)	3 (1.08)	1 (0.44)	1 (0.36)
15–	254 (83.28)	256 (78.05)	226 (76.61)	200 (76.05)	249 (78.55)	205 (75.37)	201 (72.04)	215 (69.35)	209 (71.33)	189 (67.99)	160 (71.11)	190 (68.59)
60–	49 (16.07)	68 (20.73)	61 (20.68)	63 (23.95)	66 (20.82)	66 (24.26)	76 (27.24)	93 (30)	82 (27.99)	86 (30.94)	64 (28.44)	86 (31.05)
Occupation, n (%)												
Farmer	201 (65.90)	192 (58.54)	179 (60.68)	150 (57.03)	149 (47)	157 (57.72)	181 (64.87)	124 (40)	109 (37.2)	121 (43.53)	82 (36.44)	110 (39.71)
Worker	45 (14.75)	44 (13.41)	32 (10.85)	26 (9.89)	38 (11.99)	21 (7.72)	26 (9.32)	74 (23.87)	72 (24.57)	49 (17.63)	46 (20.44)	51 (18.41)
Housework and unemployment	13 (4.26)	45 (13.72)	22 (7.46)	49 (18.63)	91 (28.71)	57 (20.96)	40 (14.34)	65 (20.97)	59 (20.14)	53 (19.06)	52 (23.11)	48 (17.33)
Retiree	8 (2.62)	7 (2.13)	12 (4.07)	7 (2.66)	4 (1.26)	7 (2.57)	12 (4.30)	9 (2.9)	8 (2.73)	10 (3.6)	8 (3.56)	26 (9.38)
Student	11 (3.61)	11 (3.35)	17 (5.76)	11 (4.18)	13 (4.10)	8 (2.94)	8 (2.87)	8 (2.58)	7 (2.39)	18 (6.47)	12 (5.33)	6 (2.17)
Others	27 (8.86)	29 (8.85)	33 (11.18)	20 (7.61)	22 (6.94)	22 (8.09)	12 (4.30)	30 (9.68)	38 (12.97)	27 (9.71)	25 (11.12)	36 (13.00)
Interval from onset to the visiting in designated hospital (day), n (%)												
0–13	185 (60.66)	185 (56.40)	160 (54.24)	138 (52.47)	185 (58.36)	170 (62.50)	160 (57.35)	167 (53.87)	121 (41.30)	130 (46.76)	107 (47.56)	140 (50.54)
≥14	120 (39.34)	143 (43.60)	135 (45.76)	125 (47.53)	132 (41.64)	102 (37.50)	119 (42.65)	143 (46.13)	172 (58.70)	148 (53.24)	118 (52.44)	137 (49.46)
Interval from visiting in designated hospital to confirmation of PTB (day), n (%)												
0–13	262 (85.90)	284 (86.59)	244 (82.71)	215 (81.75)	261 (82.33)	221 (81.25)	226 (81.00)	242 (78.06)	210 (71.67)	195 (70.14)	166 (73.78)	204 (73.65)
≥14	43 (14.10)	44 (13.41)	51 (17.29)	48 (18.25)	56 (17.67)	51 (18.75)	53 (19.00)	68 (21.94)	83 (28.33)	83 (29.86)	59 (26.22)	73 (26.35)

### 3.2. Comparison of various delays between local and migrant patients

The median patient and total delays for local TB patients notified in the Fenghua District were longer than those for migrant patients; the median health system delay did not show any statistically significant difference. Moreover, the proportions of total delay and patient delay were higher in the local population than in the migrant group, although this difference was not observed in health system delays. Details are shown in Tables 2–4.

**Table 2:** Patient delay of local and migrant TB patients from 2010 to 2021 in Fenghua District.

Year	Days (median, IQR)			Proportion (n, %)		
	Local	Migrant	*p*-value	Local	Migrant	*P*-value
2010	4 (1–12)	3 (1–6)	0.05	35 (19.13)	8 (6.56)	0.00
2011	5 (3–9)	4 (2–7)	0.01	35 (19.34)	9 (6.12)	0.00
2012	4 (2–11)	3 (1–6)	0.01	42 (22.46)	9 (8.33)	0.00
2013	6 (3–13)	5 (3–7)	0.00	38 (23.46)	10 (9.90)	0.01
2014	5 (2–13)	3 (1–7)	0.01	36 (21.82)	20 (13.16)	0.04
2015	4 (1–14)	3 (1–7)	0.03	36 (25.17)	15 (11.63)	0.00
2016	5 (1–14)	3 (1–7)	0.01	38 (28.15)	15 (10.42)	0.00
2017	10 (4–15)	4 (1–8)	0.00	50 (33.78)	18 (11.11)	0.00
2018	10 (4–17)	5 (2–13)	0.00	48 (39.34)	35 (20.47)	0.00
2019	13 (5–21)	4 (2–10)	0.00	59 (48.76)	24 (15.29)	0.00
2020	11 (4–18)	4 (1–11)	0.00	31 (38.75)	28 (19.31)	0.00
2021	8.5 (5–15)	5 (2–14)	0.01	28 (28.57)	45 (25.14)	0.54
2010–2021	6 (3–14)	4 (2–8)	0.00	476 (27.59)	236 (13.74)	0.00

**Table 3:** Health system delay of local and migrant TB patients from 2010 to 2021 in Fenghua District.

Year	Days (median, IQR)			Proportion (n,%)		
	Local	Migrant	*P*-value	Local	Migrant	*P*-value
2010	9 (2–22)	9.5 (4–31)	0.08	71 (38.80)	49 (40.16)	0.81
2011	12 (5–27)	9 (2–28)	0.21	80 (44.20)	63 (42.86)	0.81
2012	13 (4–31)	10 (3–28)	0.35	92 (49.20)	43 (39.81)	0.12
2013	10.5 (3–31)	13 (2–31)	0.99	76 (46.91)	49 (48.51)	0.80
2014	9 (3–24)	11.5 (3–30)	0.55	66 (40.00)	66 (43.42)	0.54
2015	9 (3–15)	12 (4–19)	0.11	46 (32.17)	56 (43.41)	0.06
2016	8 (1–21)	12 (3–27)	0.04	50 (37.04)	69 (47.92)	0.07
2017	10 (2–30)	14 (6–28)	0.08	59 (39.86)	84 (51.85)	0.03
2018	15 (7–23)	15 (5–35)	0.65	73 (59.84)	99 (57.89)	0.74
2019	14 (6–30)	14 (5–31)	0.77	65 (53.72)	83 (52.87)	0.89
2020	14.5 (3–21)	14 (4–31)	0.56	41 (51.25)	77 (53.10)	0.79
2021	15 (7–36)	10 (2–31)	0.01	55 (56.12)	82 (45.81)	0.10
2010–2021	11 (3–28)	12 (4–30)	0.12	774 (44.87)	820 (47.76)	0.09

**Table 4:** The total delay of local and migrant TB patients from 2010 to 2021 in Fenghua District.

Year	Days (median, IQR)			Proportion (n,%)		
	Local	Migrant	*P*-value	Local	Migrant	*P*-value
2010	18 (9–34)	16 (9–36)	0.79	112 (61.20)	68 (55.74)	0.34
2011	20 (12–35)	15 (8–34)	0.01	124 (68.51)	79 (53.74)	0.01
2012	24 (11–50)	14.5 (7–36)	0.03	127 (67.91)	59 (54.63)	0.02
2013	23 (12–44)	20 (8–37)	0.14	119 (73.46)	62 (61.39)	0.04
2014	19 (10–36)	17 (9–33)	0.46	105 (63.64)	96 (63.16)	0.93
2015	16 (10–30)	16 (7–28)	0.59	94 (65.73)	80 (62.02)	0.52
2016	18 (10–31)	17 (8–32)	0.98	87 (64.44)	89 (61.81)	0.65
2017	22 (12–43)	19 (11–33)	0.11	105 (70.95)	111 (68.52)	0.64
2018	24 (18–44)	21 (15–62)	0.26	109 (89.34)	137 (80.12)	0.03
2019	29 (19–49)	19 (13–39)	0.00	109 (90.08)	117 (74.52)	0.00
2020	25 (16–42)	19 (14–43)	0.07	70 (87.50)	110 (75.86)	0.04
2021	31 (17–56)	19 (13–38)	0.00	85 (86.73)	134 (74.86)	0.02
2010–2021	21 (13–39)	18 (10–37)	0.00	1,246 (72.23)	1,142 (66.51)	0.00

Additionally, we analyzed the diversity in patient characteristics between local and migrant patients across the three delays. For patient delay, the median delay time was longer in local than in the migrant patients except for the “0–14” age group and students. For health system delay, only patients aged >60 years presented a longer delay in the migrant group, while other variables were not identified as statistically significant. For the total delay, males, farmers, and age among the “15–59” age group had a higher delay in local than in the migrant group. This information is presented in Table 5.

**Table 5:** Delays of local and migrant TB patients from 2010 to 2021 in Fenghua District (median, IQR).

Variables	Patient delay			Health system delay			The total delay		
	Local	Migrant	*P*-value	Local	Migrant	*P*-value	Local	Migrant	*P*-value
Gender									
Male	6 (3–15)	4 (2–8)	0.00	10 (3–23)	11 (3–28)	0.32	21 (12–38)	17 (9–35)	0.00
Female	6 (2–14)	4 (2–8)	0.00	13 (4–30)	14 (5–31)	0.23	22 (13–40)	20 (12–42)	0.50
Age									
0–	8 (4–14)	5 (2–16)	0.52	13 (7–30)	12 (7–31)	0.87	23 (14–44)	26 (12–45)	0.40
15–	5 (2–13)	4 (2–8)	0.00	11 (3–25)	11 (3–27)	0.56	20 (12–36)	17 (9–35)	0.01
60–	8 (3–17)	4 (1–11)	0.00	11 (3–29)	17 (7–39)	0.00	24 (14–45)	27 (14–59)	0.78
Occupation									
Farmer	7 (2–15)	4 (1–7)	0.00	11 (3–25)	11 (4–31)	0.22	22 (13–38)	17 (9–38)	0.00
Worker	5 (3–12)	4 (2–9)	0.01	13 (5–23)	12 (3–22)	0.41	20 (12–34)	17 (10–32)	0.47
Housework and unemployment	6 (2–14)	4 (1–9)	0.00	15 (6–30)	14 (4–30)	0.68	22 (13–41)	18 (12–37)	0.53
Retiree	12 (3–23)	5 (1–13)	0.00	13 (2–43)	17 (7–36)	0.12	32 (18–75)	27 (16–50)	0.45
Student	7 (2–10)	4 (2–8)	0.13	7 (2–19)	11 (2–29)	0.56	17 (8–30)	19 (8–38)	0.54
Others	6 (3–12)	4 (2–9)	0.02	10 (2–30)	8 (2–28)	0.69	19 (11–41)	18 (10–37)	0.61

### 3.3. Univariate/multiple-factor logistic regression analysis identified influencing factors

Considering the higher disease burden caused by delays in the older adults, we collected information on this specific group using detailed health records from BPHSP. A total of 202 patients aged >60 years were included for further analysis. Univariate and multiple-factor analyses were performed to identify the significant variables between patient and total delays. Regarding patient delay, females [odds ratio (cOR): 1.93, 95% confidence interval (CI): 1.07–3.48], educational level of elementary school and middle school (cOR: 0.23, 95% CI: 0.06–0.84) showed statistically differences; however, patients without diabetes showed a higher delay for multiple-factor analysis (aOR: 2.12, 95% CI: 1.02–4.41). Furthermore, only the education level of elementary school and middle school presented a low total delay for the univariate (cOR: 0.22, 95% CI: 0.06–0.82) and multivariate analysis (aOR: 0.21, 95% CI: 0.05–0.83). This information is presented in Tables 6, 7.

**Table 6:** The influencing factors analysis of patient delay among the older persons.

Variable	No. of delay	No. of cases	*p-*value	Crude odds ratio (95% CI)	Adjusted odds ratio (95% CI)
Age			0.40		
60–	59	114		1 (Reference)	1 (Reference)
70–	34	66		0.99 (0.54–1.82)	1.09 (0.56–2.11)
80–	8	22		0.53 (0.21–1.37)	0.60 (0.21–1.76)
Gender			0.03		
Male	58	131		1 (Reference)	1 (Reference)
Female	43	71		1.93 (1.07–3.48)	2.07 (0.99–4.32)
Educational level			0.08		
Illiteracy	12	15		1 (Reference)	1 (Reference)
Elementary school and middle school	83	174		0.23 (0.06–0.84)	0.31 (0.08–1.21)
High school and above	6	13		0.21 (0.04–1.14)	0.24 (0.04–1.39)
Marital status			0.52		
Married	97	192		1 (Reference)	1 (Reference)
Others	4	10		0.65 (0.18–2.39)	0.68 (0.17–2.81)
Occupation			0.45		
Farmer	91	185		1 (Reference)	1 (Reference)
Others	10	17		1.48 (0.54–4.04)	1.48 (0.54–4.04)
Smoking status			0.48		
Current smoker	11	25		1 (Reference)	1 (Reference)
Prior smoker	8	20		0.61 (0.24–1.57)	0.63 (0.20–2.04)
Never smoker	82	157		0.72 (0.31–1.68)	0.84 (0.31–2.34)
Drinking status			0.93		
Non-drinkers	79	159		1 (Reference)	1 (Reference)
Occasional drinkers	4	7		1.35 (0.29–6.23)	2.89 (0.45–18.44)
Frequent drinkers	18	36		1.01 (0.49–2.09)	1.41 (0.59–3.41)
BMI			0.84		
<18.5	17	36		1 (Reference)	1 (Reference)
18.5–	69	134		1.19 (0.57–2.48)	1.29 (0.55–3.02)
≥24	15	32		0.99 (0.38–2.56)	1.08 (0.37–3.21)
Physical exercise			0.46		
Yes	38	71		1 (Reference)	1 (Reference)
No	63	131		0.81 (0.45–1.44)	0.93 (0.49–1.76)
Treatment category			0.24		
New	97	190		1 (Reference)	1 (Reference)
Retreated	4	12		0.48 (0.14–1.65)	0.42 (0.11–1.59)
Diabetes			0.11		
Yes	20	50		1 (Reference)	1 (Reference)
No	81	152		1.72 (0.77–1.89)	2.12 (1.02–4.41)
Bacteriological results			0.42		
Negative	38	84		1 (Reference)	1 (Reference)
Positive	63	118		1.20 (0.89–3.28)	1.24 (0.75–2.06)
Medical insurance			0.62		
Urban residents	87	169		1 (Reference)	1 (Reference)
Others	14	33		0.89 (0.58–1.39)	0.88 (0.55–1.42)

**Table 7:** The influencing factors analysis of total delay among the older persons.

Variable	No. of delay	No. of cases	*P-*value	Crude odds ratio (95% CI)	Adjusted odds ratio (95% CI)
Age			0.39		
60–	58	114		1 (Reference)	1 (Reference)
70–	35	66		1.09 (0.59–2.00)	1.32 (0.68–2.56)
80–	8	22		0.55 (0.22–1.42)	0.60 (0.20–1.73)
Gender			0.19		
Male	61	131		1 (Reference)	1 (Reference)
Female	40	71		1.48 (0.83–2.65)	1.56 (0.76–3.19)
Educational level			0.07		
Illiteracy	12	15		1 (Reference)	1 (Reference)
Elementary school and middle school	82	174		0.22 (0.06–0.82)	0.21 (0.05–0.83)
High school and above	7	13		0.29 (0.06–1.55)	0.29 (0.05–1.67)
Marital status			0.52		
Married	97	192		1 (Reference)	1 (Reference)
Others	4	10		0.65 (0.18–2.39)	0.68 (0.17–2.78)
Occupation			0.80		
Farmer	93	184		1 (Reference)	1 (Reference)
Others	8	18		0.88 (0.33–2.38)	0.70 (0.22–2.23)
Smoking status			0.86		
Current smoker	13	25		1 (Reference)	1 (Reference)
Prior smoker	11	20		1.27 (0.50–3.23)	1.80 (0.58–5.61)
Never smoker	77	157		1.13 (0.48–2.62)	1.61 (0.59–4.41)
Drinking status			0.88		
Non-drinkers	80	159		1 (Reference)	1 (Reference)
Occasional drinkers	4	7		1.35 (0.29–6.23)	1.08 (0.18–6.52)
Frequent drinkers	18	36		1.01 (0.49–2.09)	0.73 (0.31–1.76)
BMI			0.93		
<18.5	18	36		1 (Reference)	1 (Reference)
18.5–	66	134		0.97 (0.47–2.03)	1.22 (0.53–2.80)
≥24	17	32		1.13 (0.44–2.94)	1.50 (0.51–4.36)
Physical exercise			0.88		
Yes	36	71		1 (Reference)	1 (Reference)
No	65	131		1.04 (0.59–1.86)	0.93 (0.49–1.74)
Treatment category			0.55		
New	94	190		1 (Reference)	1 (Reference)
Retreated	7	12		1.43 (0.44–4.66)	1.35 (0.38–4.85)
Diabetes			0.33		
Yes	22	50		1 (Reference)	1 (Reference)
No	79	152		1.38 (0.72–2.62)	1.35 (0.66–2.76)
Bacteriological results			0.30		
Negative	37	84		1 (Reference)	1 (Reference)
Positive	64	118		1.27 (0.81–1.99)	1.34 (0.82–2.19)
Medical insurance					
Urban residents	87	169		1 (Reference)	1 (Reference)
Others	14	33		0.85 (0.55–1.32)	0.90 (0.56–1.43)

## 4. Discussion

Patient, health system, and total delays, have been widely used to assess the effectiveness of existing PTB control programs and identify programmatic impediments during PTB diagnosis (10). Delays in diagnosing PTB are a common challenge for all low- and middle-income countries, accounting for a large proportion of the global burden (4). Passive case-finding strategies lead to poor prognosis and high disease transmission. The WHO guidelines recommend that patients coughing for more than 2 weeks should be screened for tuberculosis (14). However, in China, the median delay in diagnosis is 29 days, which does not meet the WHO requirements for timely diagnosis (15). To achieve the goal of ending tuberculosis by 2035, some interventions and implementations, such as mass screening of target populations and health checkups, have been performed to achieve timely diagnosis and sufficient treatment.

Between 2010 and 2021, 3,442 PTB cases were reported in the Fenghua District. Most reported PTB cases were concentrated in young and middle-aged populations, and the overall notification rate was decreasing. This could be attributed to a range of strategies and actions implemented to prevent and control PTB and the government’s recent dedication and investment in public health (16). However, the proportion of cases in individuals aged >60 years is rapidly increasing, indicating an urgent need to address PTB in aging populations. It is estimated that by 2050, older adults population aged ≥65 years in China will account for a quarter of the total population (17). Additionally, an estimated 90% of TB cases will result from the reactivation of latent TB infection acquired earlier in life, rather than from recent transmission among the older adults (18–20). Therefore, attention should be paid to these issues, and more efficient and effective measures, such as treating latent infections and addition of specific nutrition, should be investigated in this group.

The proportion of migrant patients in the Fenghua District was 49.88%, comparable to the composition of PTB cases in Shanghai City (21) and higher than the average at the Zhejiang provincial level (22), reflecting the huge PTB risk caused by the trend of population mobility in China. Moreover, approximately 244 million immigrants in China (23) brought about new changes to TB control.

Our study revealed that the median patient and total delays were longer in local patients than in the migrant patients. This difference may be attributed to the “healthy migrant effect” and the “salmon bias” hypotheses (24, 25). The migrants in our study were younger than the local population and had a higher level of health awareness, potentially motivating them to proactively seek medical attention. Additionally, they tended to visit higher-level hospitals for medical treatment, reducing the likelihood of passive TB case detection. There were also comparatively longer delays in the older migrant population. This may be because that the local health education and public health services were not accessible to migrant patients, and the older adults also had lower health knowledge of PTB, leading to neglect of suspected symptoms. For the student population, implementing a daily health checkup would shorten patient delays in public schools, while students from migrant groups commonly studied in private schools might be some insufficient. Therefore, more attention should be paid to student populations during daily health examinations.

Considering the completeness of health data in the local older population, we conducted further analyses to identify the factors contributing to delays. Previous studies have investigated the correlation between delays and sex; however, the results have been inconsistent. For instance, Kee et al. observed that women had a significantly longer total delay than men in Malaysia (26); however, some studies conducted in Eastern Mediterranean countries did not observe any sex differences in total delay (27). Furthermore, our findings revealed that the proportion of females with delays was higher than that of males. This may be because of the physiological and psychological characteristics of women and men and differences in social division (28). Women generally have a lower socioeconomic status and are more likely to delay before seeking treatment after experiencing TB-related clinical symptoms related to TB (29, 30). Additionally, owning to their susceptibility and physiological differences, female patients may also experience milder symptoms than male patients, contributing to patient delays (31). Furthermore, our study observed that patients with primary and secondary education experienced lower patient and total delays than illiterate patients. This may be attributed to the fact that illiteracy can limit patients’ access to written health education and promotional materials, typically used to raise public awareness about TB (9, 10). This finding highlighted the importance of developing audiovisual and graphical promotional materials for health education, especially for illiterate patients. However, due to the limited sampling size of people with an education level of high school and above, the homologous finding maybe affected. Furthermore, the results showed that patients without diabetes also had a longer patient delay than those with diabetes, potentially as patients with diabetes were commonly followed up by specific physicians in the local community health center in each quarter, which could improve the early detection rate.

Our study had some limitations. First, the time of onset of symptoms and first visit of patients with TB were based on self-reports; therefore, there may have been recall bias. Additionally, this study was conducted in only one county within eastern China; thus, the findings only depict the features of that area. Finally, previous studies had inconsistent criteria regarding delays, using different cutoff points to define acceptable and long delays. Our study used the median as the cutoff point for the delay, which resulted in a lack of comparability with studies that used other classification methods. Nevertheless, our study used a substantial sample size and integrated monitoring data and residents’ health records, which enhanced the authenticity and completeness of the results.

## 5. Conclusion

The delay in identifying TB cases among migrants was lower than that among the local population in the Fenghua District, which may be related to the “healthy migrant effect.” Furthermore, our research highlighted that women, illiterate people, and people without diabetes are key groups in reducing delays among the older adults. Therefore, future research should focus on raising health awareness among key populations, providing accessible health services, and reducing the time from symptom onset to a definitive diagnosis.

## Data availability statement

The original contributions presented in the study are included in the article/supplementary material, further inquiries can be directed to the corresponding authors.

## Ethics statement

This study was approved by the Ethics Committee of the Zhejiang Provincial Center for Disease Control and Prevention. Written informed consent from the patients was not required to participate in this study in accordance with the national legislation and the institutional requirements.

## Author contributions

KL, RG, and QW: conception and design. DL, YZ, and ZS: study management. WF and DL: analysis of the data. KL, RG, and BC: interpretation of study results. KL, RG, and WF: initial drafting the manuscript for important intellectual content. BC and QW: review and editing of manuscript. KL, RG, DL, YZ, ZS, BC, WF, and QW: approval of the final version to be published. All authors contributed to the article and approved the submitted version.

## Funding

This study was supported by the National-Zhejiang Health Commission Major S&T Project (Grant no. WKJ-ZJ-2118), Zhejiang Provincial Medical and Health Project (Grant nos. 2021KY618 and 2020KY520), and the Social Development Scientific Research Project of Fenghua District (Grant no. 202209208).

## Conflict of interest

The authors declare that the research was conducted in the absence of any commercial or financial relationships that could be construed as a potential conflict of interest.

## Publisher’s note

All claims expressed in this article are solely those of the authors and do not necessarily represent those of their affiliated organizations, or those of the publisher, the editors and the reviewers. Any product that may be evaluated in this article, or claim that may be made by its manufacturer, is not guaranteed or endorsed by the publisher.

## Footnotes

^1^https://www.r-project.org/
